# Electrophysiological Evidence for Intrinsic Pacemaker Currents in Crayfish Parasol Cells

**DOI:** 10.1371/journal.pone.0146091

**Published:** 2016-01-14

**Authors:** DeForest Mellon

**Affiliations:** Department of Biology, University of Virginia, Charlottesville, Virginia, United States of America; University of South Carolina, UNITED STATES

## Abstract

I used sharp intracellular electrodes to record from parasol cells in the semi-isolated crayfish brain to investigate pacemaker currents. Evidence for the presence of the hyperpolarization-activated inward rectifier potassium current was obtained in about half of the parasol cells examined, where strong, prolonged hyperpolarizing currents generated a slowly-rising voltage sag, and a post-hyperpolarization rebound. The amplitudes of both the sag voltage and the depolarizing rebound were dependent upon the strength of the hyperpolarizing current. The voltage sag showed a definite threshold and was non-inactivating. The voltage sag and rebound depolarization evoked by hyperpolarization were blocked by the presence of 5–10 mM Cs^2+^ ions, 10 mM tetraethyl ammonium chloride, and 10 mM cobalt chloride in the bathing medium, but not by the drug ZD 7288. Cs^+^ ions in normal saline in some cells caused a slight increase in mean resting potential and a reduction in spontaneous burst frequency. Many of the neurons expressing the hyperpolarization-activated inward potassium current also provided evidence for the presence of the transient potassium current I_A_, which was inferred from experimental observations of an increased latency of post-hyperpolarization response to a depolarizing step, compared to the response latency to the depolarization alone. The latency increase was reduced in the presence of 4-aminopyridine (4-AP), a specific blocker of I_A_. The presence of 4-AP in normal saline also induced spontaneous bursting in parasol cells. It is conjectured that, under normal physiological conditions, these two potassium currents help to regulate burst generation in parasol cells, respectively, by helping to maintain the resting membrane potential near a threshold level for burst generation, and by regulating the rate of rise of membrane depolarizing events leading to burst generation. The presence of post-burst hyperpolarization may depend upon I_A_ channels in parasol cells.

## Introduction

Periodic electrical activity in neurons and other rhythmic cells largely depends for its expression on intrinsic membrane currents. These currents are responsible for the oscillations in membrane potential, which act to generate the cellular output and also are at least partially responsible for activation of the currents themselves. Thus, in many tissues, the extent and timing of the membrane oscillations are orchestrated by voltage-gated ion channels, whose collective thresholds and activation and inactivation kinetics, though often modulated by synaptic or other neurohumoral agents, uniquely define the output properties of the cell. Examples include the cardiac rhythms in both vertebrates [1] and invertebrates [2, 3, 4, 5, 6];, rhythmically-active neurosecretory cells [7], mammalian respiratory rhythms [8, 9], and rhythmic activities of the crustacean gut [10,11 12, 13]. Motor neurons of the pyloric network in the lobster and crab stomatogastric ganglion (STG) are readily-studied model systems to investigate synaptic interactions among rhythmically active neurons, modulation of network and cellular properties by naturally occurring biogenic amines and peptides, and intrinsic properties of the motor neurons themselves [14, 15, 16] In the dorsal gastric motor neuron of the crab STG, for example, the currents believed to modulate potential changes leading up to plateau potentials that underlie a spike burst envelope are a transient, depolarization-activated and inactivated potassium current (I_A_) with relatively fast kinetics and a hyperpolarization-activated, non-inactivating cation current (I_h_) with very slow kinetics [17,11].

Parasol cells are multimodal interneurons that have been described in the lateral protocerebrum of the brains of crayfishes and the lobster *Homarus americanus* [18, 19, 20]. Parasol cells receive periodic excitatory input from unidentified centers elsewhere within the brain; in response to this ongoing synaptic activity, they spike periodically and occasionally may generate single spike bursts [21, 22, 23]. When the periodic excitatory synaptic activity is blocked by perfusing the preparation with low-calcium, high-magnesium saline, however, parasol cells depolarize and generate continuous spike trains [22] Thus, crayfish parasol cells, while exhibiting periodic activity in the form of externally-imposed background synaptic potentials, are not rhythmically activated by intrinsic ionic currents, as are, for example, cells of the crustacean stomatogastric ganglion and the mammalian respiratory kernel. Parasol cells also receive direct multimodal synaptic input from the accessory lobes of the deutocerebrum via axons that run within the lateral protocerebral tract within the eyestalks [18]. In response to especially strong sensory stimulation, parasol cells generate trains of 5–6 impulse bursts having interburst intervals similar to, or slightly less than, the period of the background activity [22, 23, 18, 19, 24] and which, from the constancy of intra-burst spike number and frequency trajectory, appear to be generated by underlying plateau potentials [24]. In view of the rich representation of the voltage-dependent membrane currents I_h_ and I_A_ in other rhythm-generating vertebrate and invertebrate neural systems, I investigated the possibility that these two pacemaker currents are present in the periodically active parasol cells of the crayfish brain. The current findings provide evidence that a hyperpolarization-activated inward rectifier potassium current, I_irk_ (but not I_h_) and I_A_ both exist in many if not most crayfish parasol cells. It is suggested that, in association with the periodic synaptic input characteristic of crustacean parasol cells, both currents may help to regulate, respectively, the resting potential level and the burst-generating properties of these neurons.

## Materials and Methods

**Fig 1:**
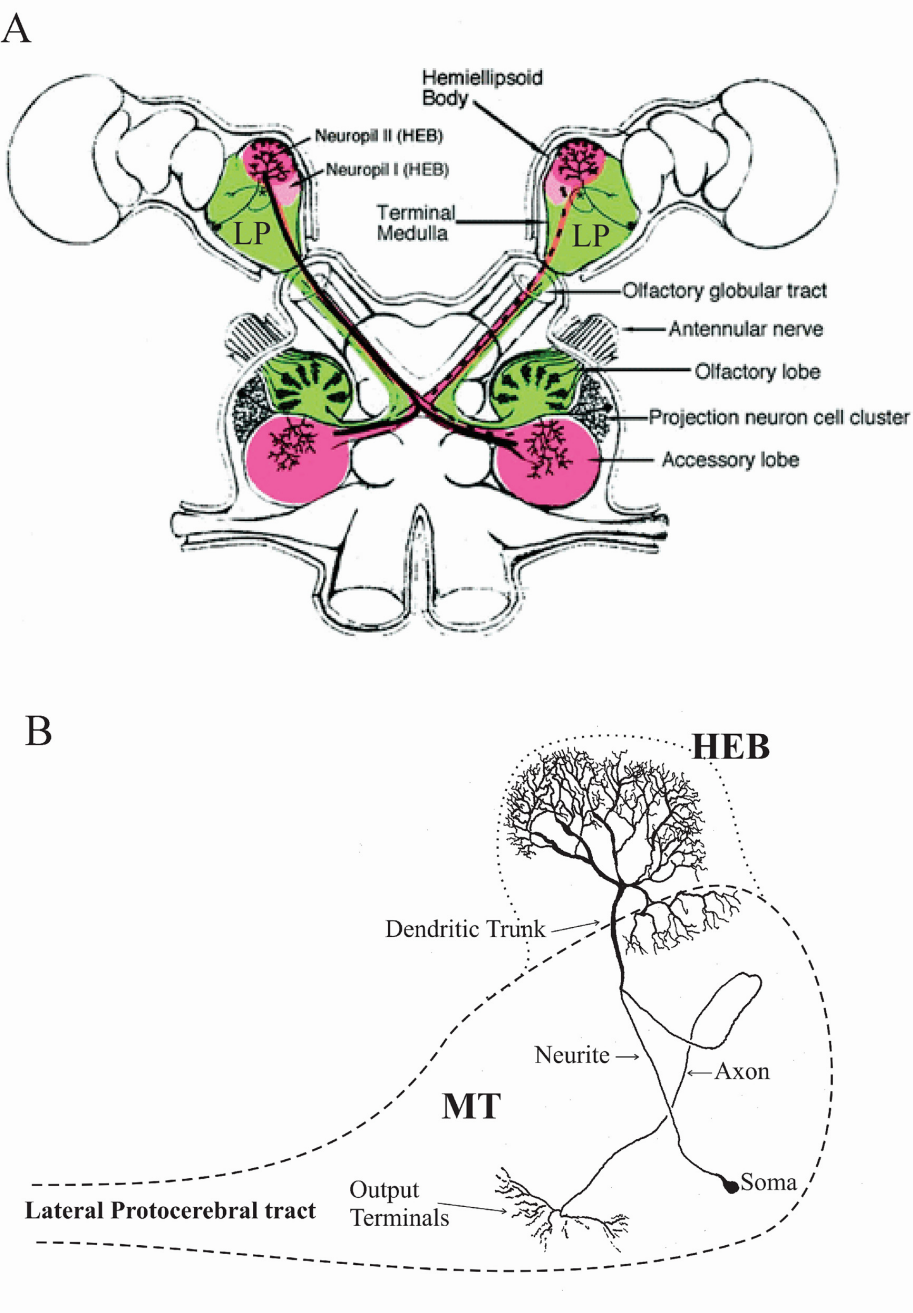
Diagrams of crayfish brain and lateral protocerebrum. A, Dorsal view of the crayfish brain indicating major deutocerebral regions (shading) and the neural structures within the optic cups. Parasol cells in the hemiellipsoid body of the lateral protocerebrum (LP) are major targets for projection neurons from the accessory lobes. Projection neuron axons run within the olfactory globular tract, a subdivision of the lateral protocerebral tract (From [44] with permission). B, Position of various morphological regions of the parasol cells within the lateral protocerebrum. A parasol cell is shown within the hemiellipsoid body/ *medulla terminalis* complex. Intracellular recordings were made from large dendritic branches within the hemiellipsoid body (HEB). The *medulla terminalis* (MT) constitutes the major structure within the lateral protocerebrum.

Fig 1A & 1B are diagrams, respectively, of the crayfish brain and the location of parasol cells within the lateral protocerebrum. The hemiellipsoid body (HEB) is a bilobed protrusion from the lateral protocerebrum, adjacent to a region referred to as the *medulla terminalis*. Each lobe of the HEB houses the extensive dendritic arbors of parasol cells, whose branches form the major targets of presynaptic axons within the lateral protocerebral tract. The specific neuronal targets of the parasol cell axons have not been identified, but their terminals are clearly located within a region of the ventral *medulla terminalis*. While some initial electrophysiological observations of parasol cell activity were made with the lobster *Homarus americanus*, all experimental procedures involving I_irk_ and I_A_ were performed with the crayfish *Procambarus clarkii* Girard, which were obtained from a supplier (Atchafalaya Biological Supply) in Raceland, Louisiana. Crayfish were kept in large (350 liter) tubs of circulating, filtered fresh water at a temperature of 18°-20°C until used. They were fed on blackworms twice a week. The culture room was on a 12:12 L:D light cycle.

**Fig 2:**
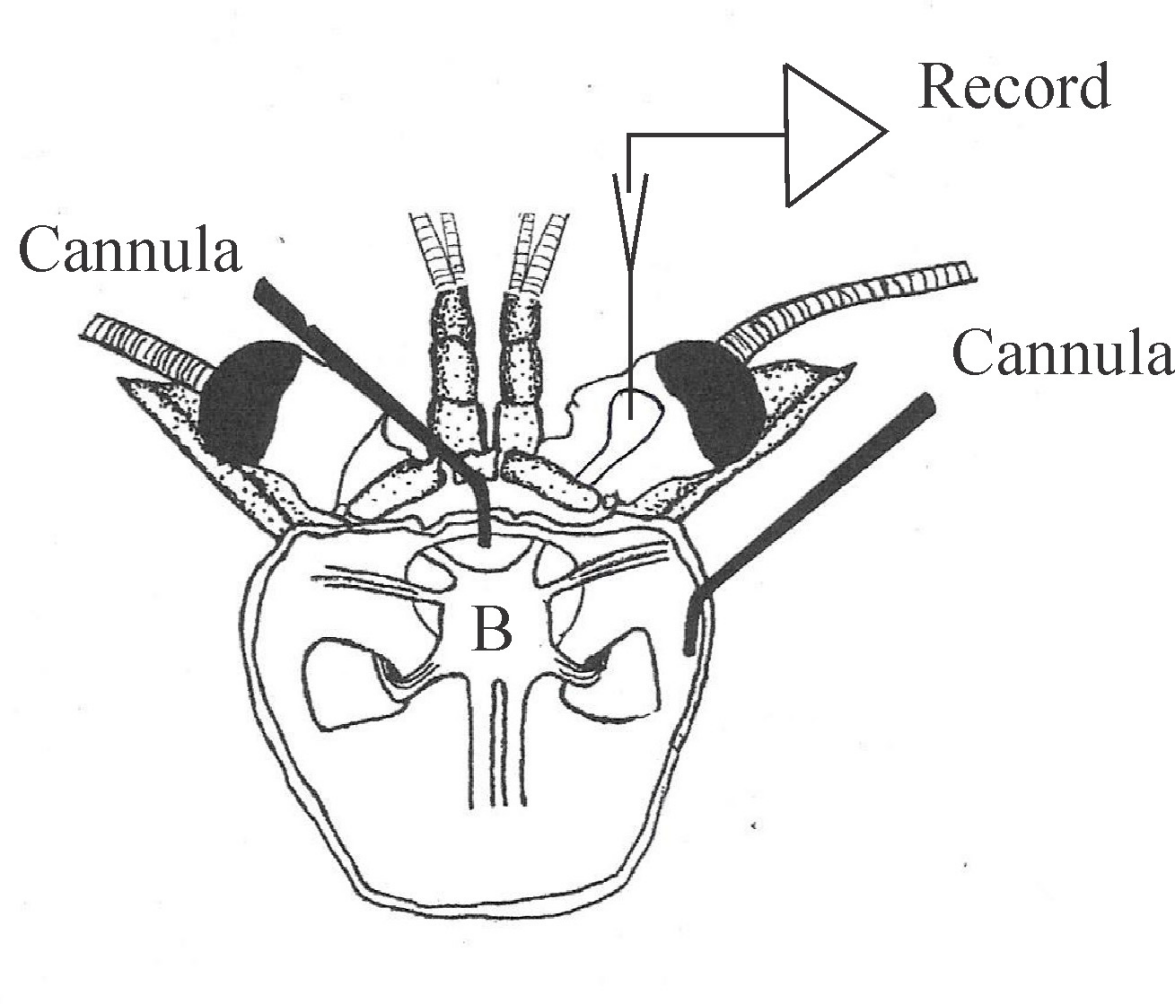
Isolated crayfish brain set-up. Diagram of the isolated crayfish head preparation used in the present study. The brain, B, is indicated within the head capsule. Cannulas perfuse saline through the median and lateral cephalic arterial systems of the head. The lateral protocerebrum is indicated at its normal position within the right eyestalk, with covering structures removed. (Modified from [45]; used with permission).

Fig 2 is a diagram of the isolated crayfish head preparation used in this study. Prior to dissection, crayfish were placed on crushed ice for 10'-15'. They were quickly decapitated by cutting around the exoskeleton just anterior to the cervical groove (for details, see [22]. The rostrum was then carefully removed, and the head capsule was pinned to a Sylgard platform within a recording chamber. The right-hand eyecup was stabilized and secured to the chamber wall with a short length of heat shrink tubing. The two major arterial systems of the isolated heads then were quickly cannulated and flushed with chilled (15°C) oxygenated crayfish saline having the following composition, in mM/L: NaCl, 205; KCl, 5.4; CaCl_2_ 2H_2_O, 13.6; MgCl_2_ 7H_2_O, 2.7, and NaHCO_3_, 2.4. The pH of the saline was adjusted to 7.4 with HCl.

A window was cut in the dorsal exoskeleton of the right eyecup, and muscles overlying the neural axis were removed by microdissection. The connective tissue sheath surrounding the *terminal medulla* and HEB was torn with forceps and further dissected with microscissors. Loose glial tissue and hemolymph around the HEB were gently washed away with a saline jet using a tuberculin syringe.

Observations with lobster parasol cells were made while I was a guest in the laboratory of Dr. Jelle Atema at Marine Biological Laboratory in Woods Hole, Massachusetts. Animals were housed in large tanks of continuous-flow natural seawater until used. Lobster isolated heads were prepared similarly to those of crayfish, in an appropriately larger recording chamber, and using a saline with the following composition: NaCl, 472; KCl 10; CaCl_2_ 2H_2_O, 16; MgCl_2_ 6H_2_O; tris base, 10; glucose, 11. The pH of the solution was adjusted to 7.4 with 0.1 M maleic acid.

Basal branches of the dendritic trees of parasol cells within the HEB [24] were penetrated with sharp microelectrodes filled with 3M KAc and having resistances of 120–200 MΩ. These high resistance electrodes permitted long-term recordings from basal branches and a minimum of injury during initial penetration. Unfortunately, they precluded even single-electrode voltage clamp studies of membrane currents, which in any case would have been confounded by difficulties in obtaining reliable space clamp conditions in the extensive, electrically porous dendritic tree of parasol cells.

Microelectrodes were connected through a salt bridge to one input stage of an Axoclamp 2B amplifier (Molecular Devices, Sunnyvale, CA), operated in bridge mode. When required, current was passed through the recording electrode using the bridge circuit and controlled by pClamp software (Axon Instruments Inc.). Bridge balance was effected after cell penetration by eliminating the DC voltage shift in a 600 μsec, 0.1 nA pulse repeated at 10 Hz. In practice, this procedure was usually not necessary, since the membrane time constant of the parasol cell basal branch response to injected current ranges up to hundreds of milliseconds, and the comparatively much more rapid voltage drop across the electrode could be balanced out by eye. All recorded data were acquired and stored using the pClamp software. Drugs and modified saline solutions were made up just prior to use and were applied to the preparation using the same pressurized delivery system as normal saline and were chilled to the same temperature. Wash-in of solutions usually took 3–5 minutes after switching on solution reservoirs. Measurement of drug effects began 5 minutes after the switch. All data shown were from preparations in which the drug effects were reversed in normal saline, although this sometimes took longer than an hour to occur. The delays to first spike following depolarizing steps, with and without a hyperpolarizing prepulse and in the presence or absence of 4-AP, were normalized, the means and standard deviations from each of 10 trials at each current level were calculated, and the data from separate treatments were compared using a one-way ANOVA program found in Origin 8.5 software (OriginLab Corp., Northampton MA 01060 USA).

**Composition of experimental bathing media used**:
Normal saline (as above)
Normal saline + 10 mM CsCl
Normal saline + 10 mM CoCl_2_
Normal saline + 10 mM TEA-Cl
Normal saline + 100 μM ZD 7288
5 x10^-7^ TTX in normal saline
5 x10^-7^ TTX in normal saline + 10 mM CsCl
Normal saline + 10mM CsCl + 1mM 4-AP

## Results

**Fig 3:**
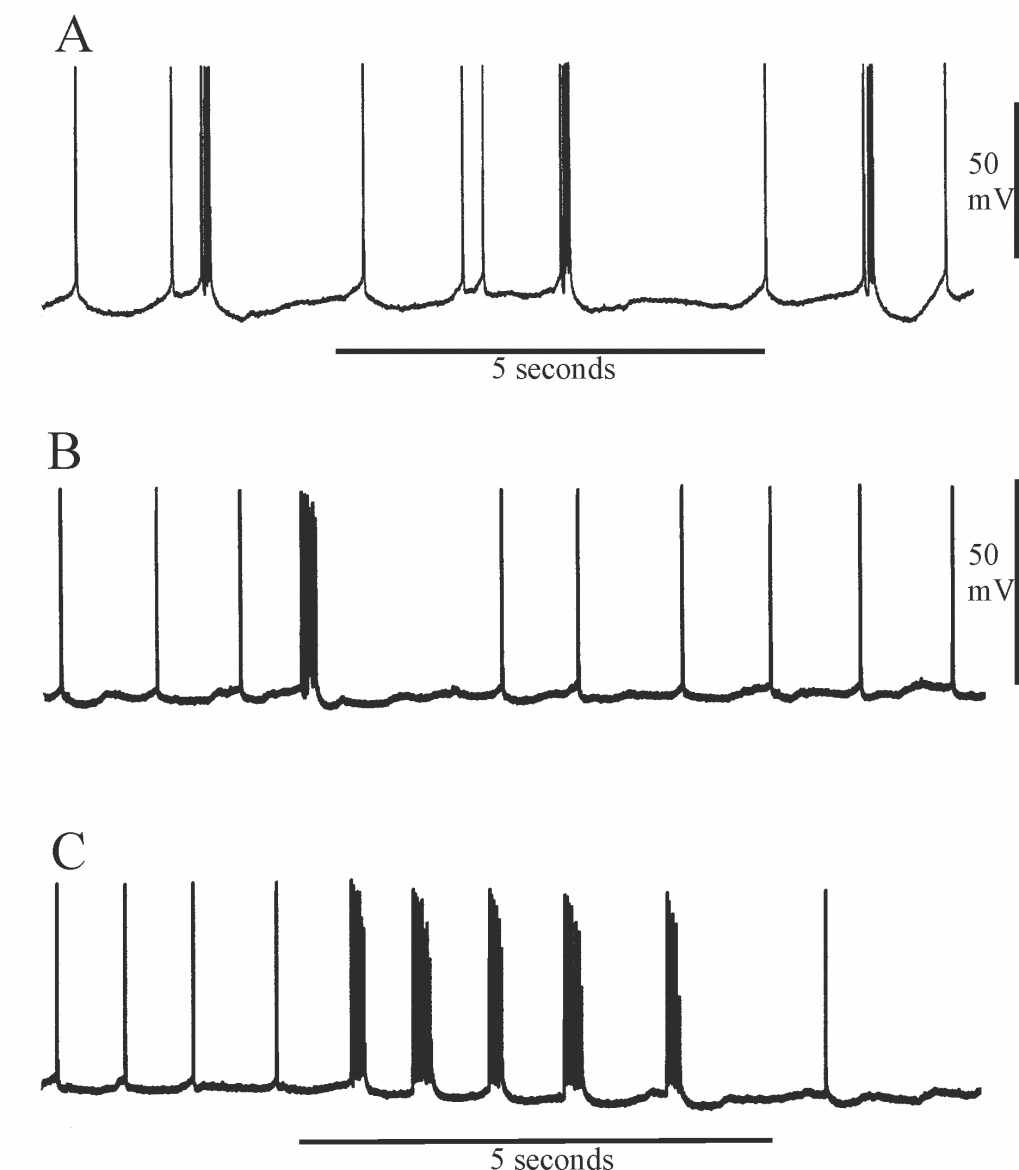
Background synaptic activity in lobster and crayfish parasol cells. A, spontaneous bursting during background activity in a parasol cell from *Homarus americanus*, recurring at approximately 4-second intervals. B, irregular, occasional spontaneous bursting during background activity in a parasol cell of *P*. *clarkii* imaged on a similar time base. Note pause in background activity following the bursts in A and B. C, repetitive bursting in the same parasol cell as in B in response to a light pulse to the ipsilateral compound eye, the duration of which is approximated by the time marker below the electrical trace.

Crustacean parasol cells generate spikes and occasional spike bursts in response to periodic synaptic drive from neural sources in adjacent regions of the protocerebrum [21, 22, 23, 18, 19, 24]. Fig 3A & 3B show typical profiles of unstimulated activity in lobster and crayfish parasol cells in normal saline. In isolated head preparations, the parasol cells of *Homarus* exhibit spontaneous periodic bursts at a more regular and higher frequency than in the crayfishes *Procambarus* or *Cherax* (Fig 3B; [21, 22]. However, in both lobsters and crayfish, sensory input can evoke trains of spike bursts that have interburst intervals similar to or less than that of the periodic synaptic drive, as exemplified in Fig 3C by the response to photic stimulation of the ipsilateral compound eye in the crayfish. Furthermore, current injected into the dendritic trunk of parasol cells generates burst trains in which the intensity of the injected current can control burst number and the interburst interval [24]. There is a suggestion, therefore, that burst intervals may be partially regulated by voltage-dependent mechanisms intrinsic to the parasol cell.

## Evidence for hyperpolarization-activated inward current in parasol cells

**Fig 4:**
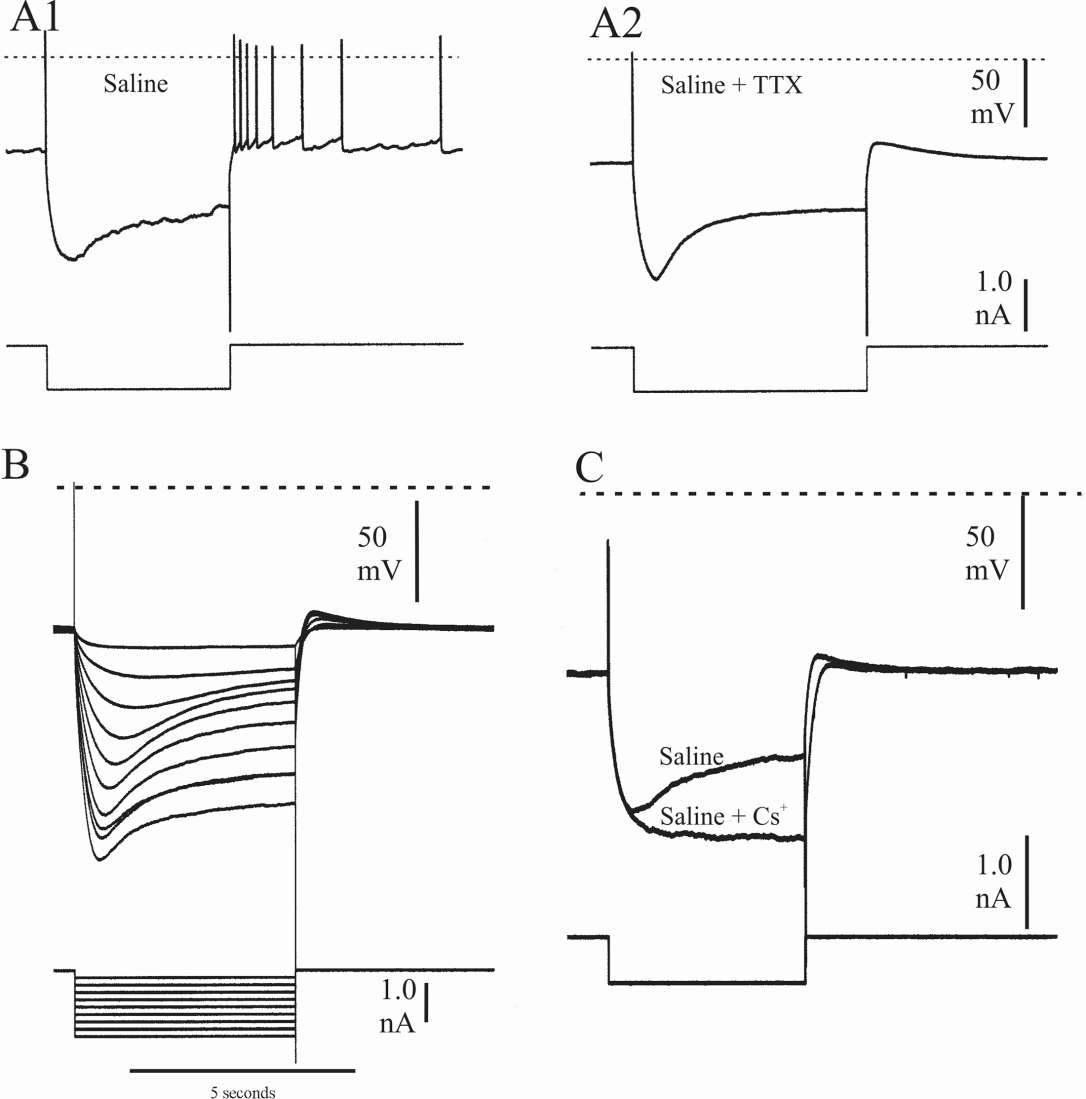
Parasol cell responses to hyperpolarizing current. A1, voltage sag and post-hyperpolarizing discharge produced in a crayfish parasol cell by a 10 sec pulse of hyperpolarizing current during background activity in normal saline. A2, sag produced by an identical current injection in the same neuron after treating the preparation with 5 x 10^−7^ M TTX. B, response of a TTX-treated crayfish parasol cell to a series of 0.2 nA steps of hyperpolarizing current. The threshold for onset of the voltage sag in this neuron was approximately 25 mV hyperpolarized to the resting potential. C, effect of the addition of 10 mM CsCl to the perfusate saline in a TTX-treated parsol cell. The voltage sag and depolarizing overshoot present in the response to a five second rectangular pulse of hyperpolarizing current in normal saline were reduced or eliminated in the saline containing Cs^+^ ions, and the recovery to resting potential level from the hyperpolarization took approximately one second longer. Broken lines in all records indicate zero membrane potential.

In more than half of the crayfish parasol cells that I analyzed by current injection (n = 23/41), evidence was obtained for the presence of a hyperpolarizing activated inward current. As shown in Fig 4A1, the presence of this current was revealed by responses to injected hyperpolarizing currents, which evoked voltage sags that were voltage dependent, rising slowly to a plateau that was maintained for as long as the current pulse lasted–more than 6 sec in this case. At the termination of the current pulse, the membrane potential overshot the resting potential in a post-hyperpolarization rebound, presumably at least partly due to a tail current. The threshold for the voltage sag was invariably high and apparently more hyperpolarized than E_K_^+^, which in crayfish neurons is above -85 mV [25]. Details of the rate of onset of the sag voltage and the depolarizing rebound were more easily examined in preparations treated with 5 x 10^−7^ M tetrodotoxin (TTX), which suppressed the background activity as well as parasol cell spiking. Fig 4A2 shows the responses of the same cell to the hyperpolarizing current step 10 min after switching to TTX saline. The voltage sag rises slowly and, in this instance, was persistent even after 10 sec. Fig 4B shows responses of another TTX-treated parasol cell to 0.2 nA steps of hyperpolarizing current. In this case, from a resting potential level of – 69 mV, the threshold voltage for the sag was about –78 mV, with higher sag amplitudes, earlier onsets, and higher levels of post-hyperpolarization depolarizing rebounds in response to greater levels of injected current. All of these responses to hyperpolarizing voltages were reduced or eliminated completely by switching to saline containing 10 mM CsCl, known to block I_h_. This can be seen in the recordings of Fig 4C. Responses of a TTX-treated parasol cell to 5-sec, 0.5 nA rectangular pulses of hyperpolarizing current before and during treatment with Cs^+^ saline are superimposed, emphasizing the reduction in sag voltage and post-hyperpolarization rebound in the presence of cesium ions. Because Cs^+^ may affect other voltage dependent cation channels [26], in two cells I also attempted to block the hyperpolarization-activated voltage sag with a more specific blocker of I_h_, ZD7288. This attempt was unsuccessful, even at concentrations 2x and 3x those effectively used in other crustacean and in mammalian preparations [27, 8]. Hyperpolarization-induced voltage sags can also indicate the presence of the hyperpolarization-activated inward rectifier potassium current, I_irk_, such as is found in neurons of the rat vagus nucleus [29] and the dorsal unpaired median neurons of the cockroach ventral nerve cord [30]. The cockroach dorsal unpaired median neurons exhibit pacemaker potentials, and I_irk_ apparently plays a role in regulating the activity profile of these cells. Unlike I_h_ studied in other crustacean neurons and in neurons of the leech heart oscillator, the hyperpolarization-activated inward rectifier potassium current I_irk_ found in the cockroach dorsal unpaired median neurons and rat vagus nucleus neurons is sensitive to divalent cations as well as to 10 mM tetraethyl ammonium ions. When I tested both 10 mM CoCl_2_ and 10 mM TEA-Cl on two crayfish parasol cell preparations that otherwise exhibited a prominent, high threshold, Cs^+^-sensitive voltage sag to hyperpolarizing currents, their presence in the saline both reversibly eliminated not only the sag but also the post hyperpolarization tail potential (experimental results documented in S1 and S2 Figs).

**Fig 5:**
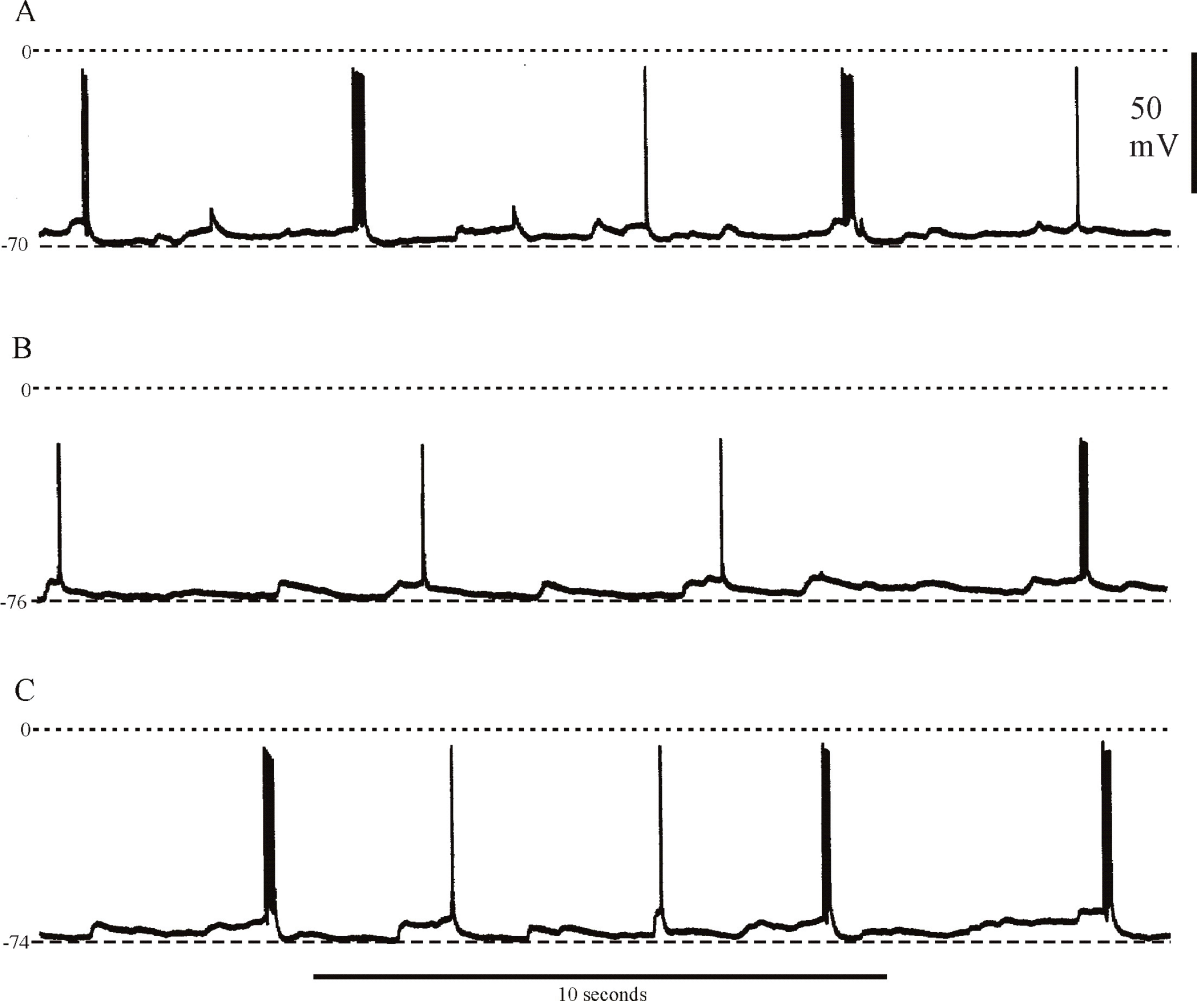
Responses of parasol cells to Cesium saline. Effects of 10 mM CsCl on the background activity of a crayfish parasol cell. A, preparation perfused with normal saline prior to application of 10 mM CsCl saline. B, after 15' in saline with Cs ^+^ ions. The maximum levels achieved by the resting membrane potential increased by 6 mV to –76 mV, and the frequency of background bursts was reduced by approximately 50%. C, recovery in normal saline after 55', by which time the resting potential had fallen to –74 mV.

The role of a hyperpolarization-activated inward current in the normal physiology of parasol cells is not intuitively apparent. One possibility is that, as in some other preparations, it could maintains a level of membrane potential that, in its absence, would be slightly higher. In parasol cells such a level of depolarization could be conducive to an enhanced generation of spikes and spike bursts by background or stimulus-generated synaptic activity. To determine whether the effects of background activity were changed in the absence of hyperpolarization-activated current, therefore, I treated cells with 10 mM CsCl in eight different preparations perfused with normal saline, and in two preparations that had been pretreated with 5 x 10^−7^ M TTX. The results of these experiments were not unequivocal; in two of the cells in normal saline, the mean resting potential was slightly increased–by 3 mV and 6 mV, respectively–and the frequency of spikes and/or bursts evoked by the background activity was reduced during the treatment, as shown by the example in Fig 5. In the six other cells, however, no measurable effects of Cs^+^ on background activity, mean resting potential or burst frequency were observed. In both instances where CsCl was added to the saline in the TTX-treated preparations, however, the mean resting potential was slightly increased (by 1.12 and 1.77 mV, respectively). In the two cells where reductions in activity were observed, there were modest increases in resting potential levels; these changes were not accompanied by any decrease in the *frequency* of background synaptic potentials, however. Another possible physiological role for a hyperpolarization-activated inward current could be in the postburst hyperpolarization that is characteristic of parasol cells [19]; Figs 3 & 5, above). The strong depolarizations associated with spike bursts and any underlying plateau potentials conceivably could lead to an inactivation of the hyperpolarization-activated inward current, providing thereby for transient membrane hyperpolarization at the end of the burst. In fact, a recent report suggests that just such a mechanism contributes to the after-hyperpolarization following strong EPSPs in CA1 hippocampal pyramidal neurons following volleys in the perforant pathway [31]. Nonetheless, in crayfish parasol cells this possibility seems unlikely in view of the high threshold required for the onset of voltage sag. Indeed, in preparations bathed in saline containing 10 mM Cs^+^ ions, in none was the post burst depression reduced or abolished by blocking the voltage sag with CsCl. Furthermore, some parasol cells that exhibited no hyperpolarization-evoked voltage sag and thus, presumably, possessed no hyperpolarization-activated inward current, still exhibited a robust post-burst depression. Therefore, the inward current generating the voltage sag appears to play no significant role in this aspect of burst physiology, and its functional significance for parasol cell electrical activity remains somewhat problematical.

### Evidence for I_A_ in parasol cells

**Fig 6:**
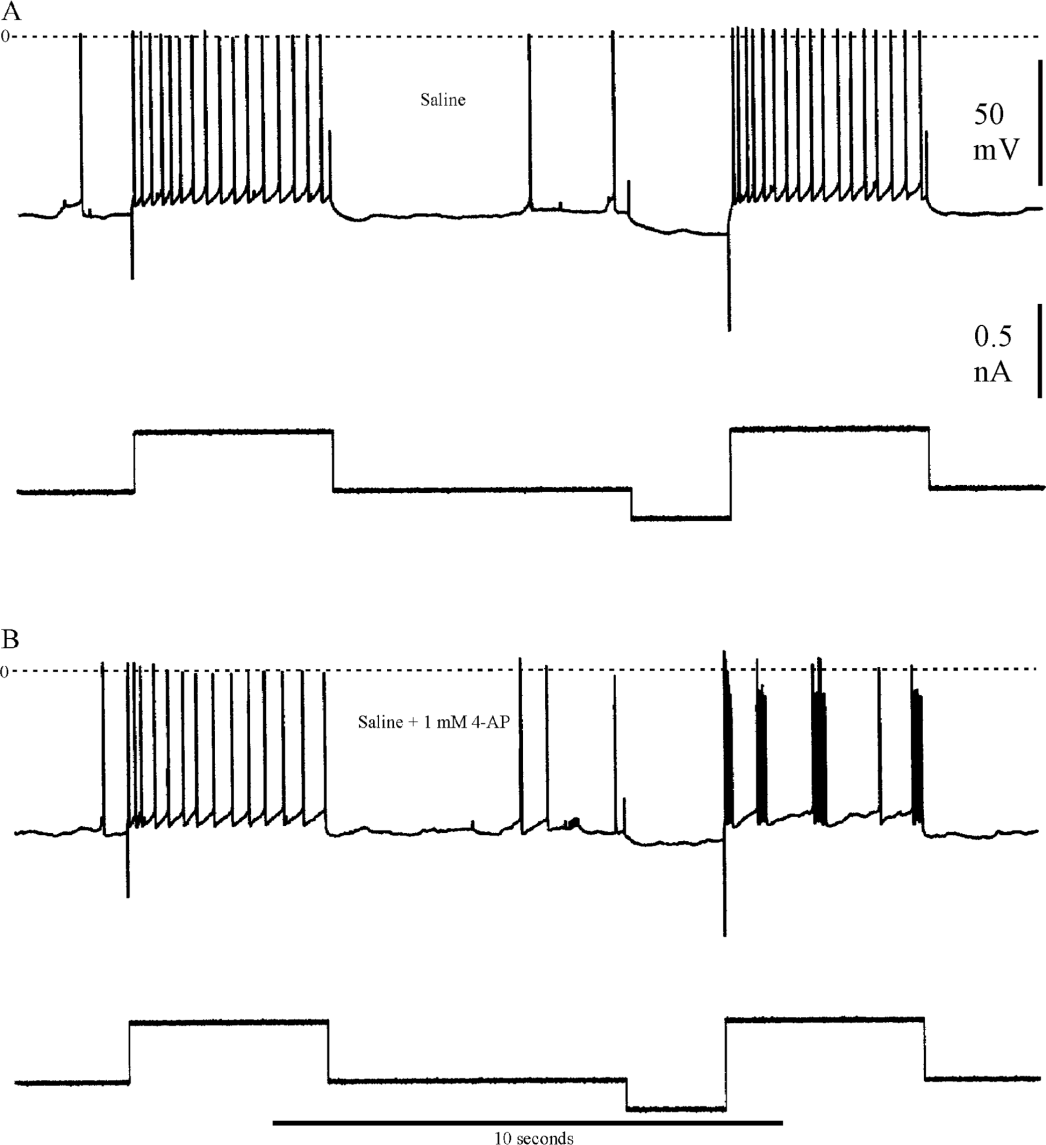
Experimental protocol to reveal I_A._ Stimulation paradigm used to reveal the presence of I_A_. A, response of a parasol cell to a 4-sec rectangular pulse of depolarizing current. After a period of no current injection, a two-second pulse of hyperpolarizing current (0.2 nA) was injected into the neuron (bottom traces), followed immediately by the previous depolarizing current injection. As discussed in the text, the hyperpolarizing prepulse delayed the onset of the spike response to the depolarization. B, treatment of the cell with 1 mM 4-AP reduced the prepulse delay and promoted bursting in the parasol cell. Zero membrane potential is indicated by the dashed lines.

**Fig 7:**
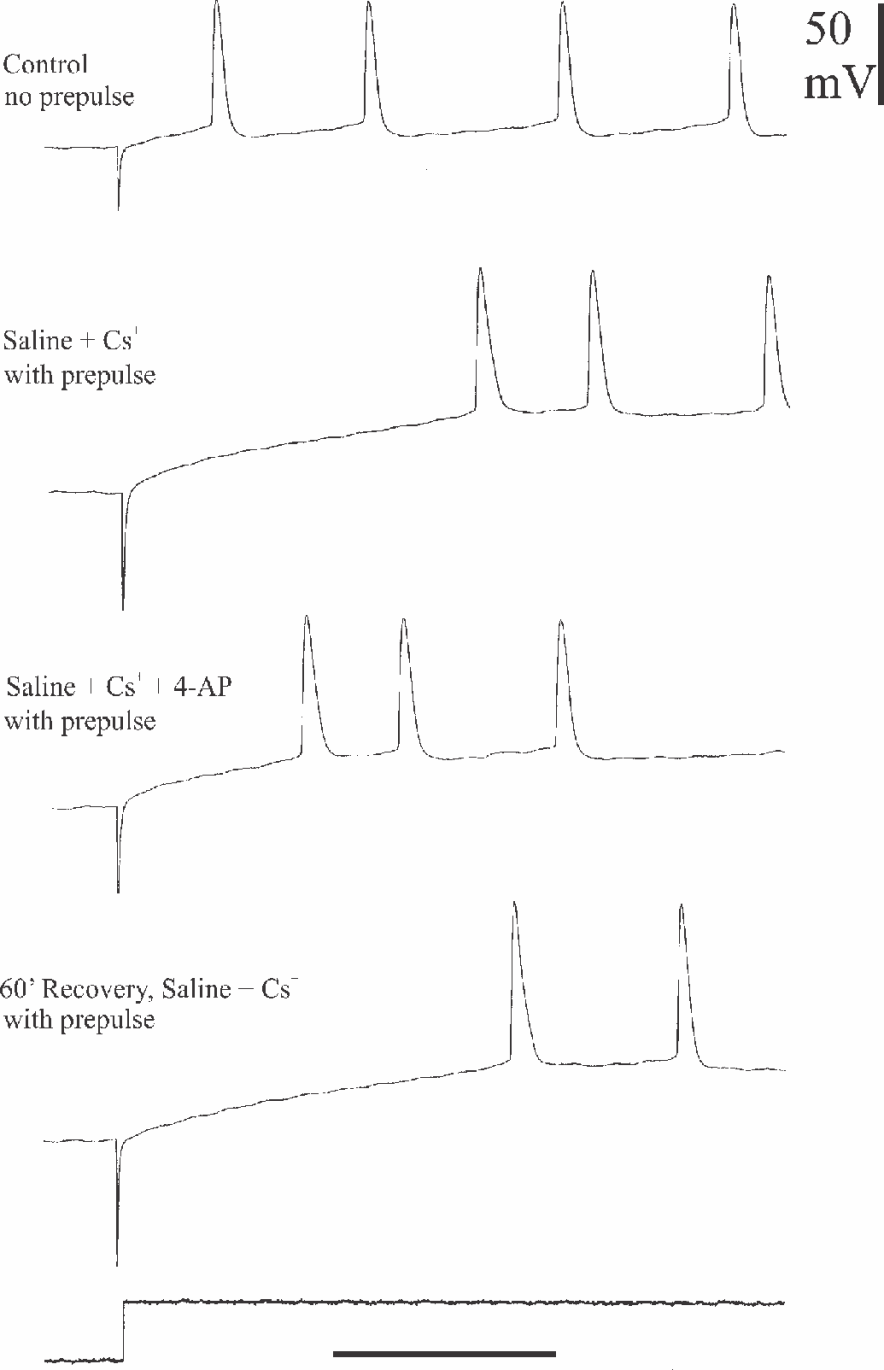
Latency to first spike electrical records. A, response of a parasol cell to a 4-sec pulse of depolarizing current (bottom trace). B-D, identical depolarizing pulses were preceded by 2-sec pulses of hyperpolarization, generated by a current level of -0.34 nAmp. In B, the delay to the first spike from the depolarizing onset was clearly increased when compared to the delay in A. In C, the preparation was treated with 1mM 4-AP for 10 min prior to the current pulses, with a concomitant reduction in delay to first spike. D shows nearly complete recovery of the delay following one hour in normal saline. Time calibration, 100 msec.

**Fig 8:**
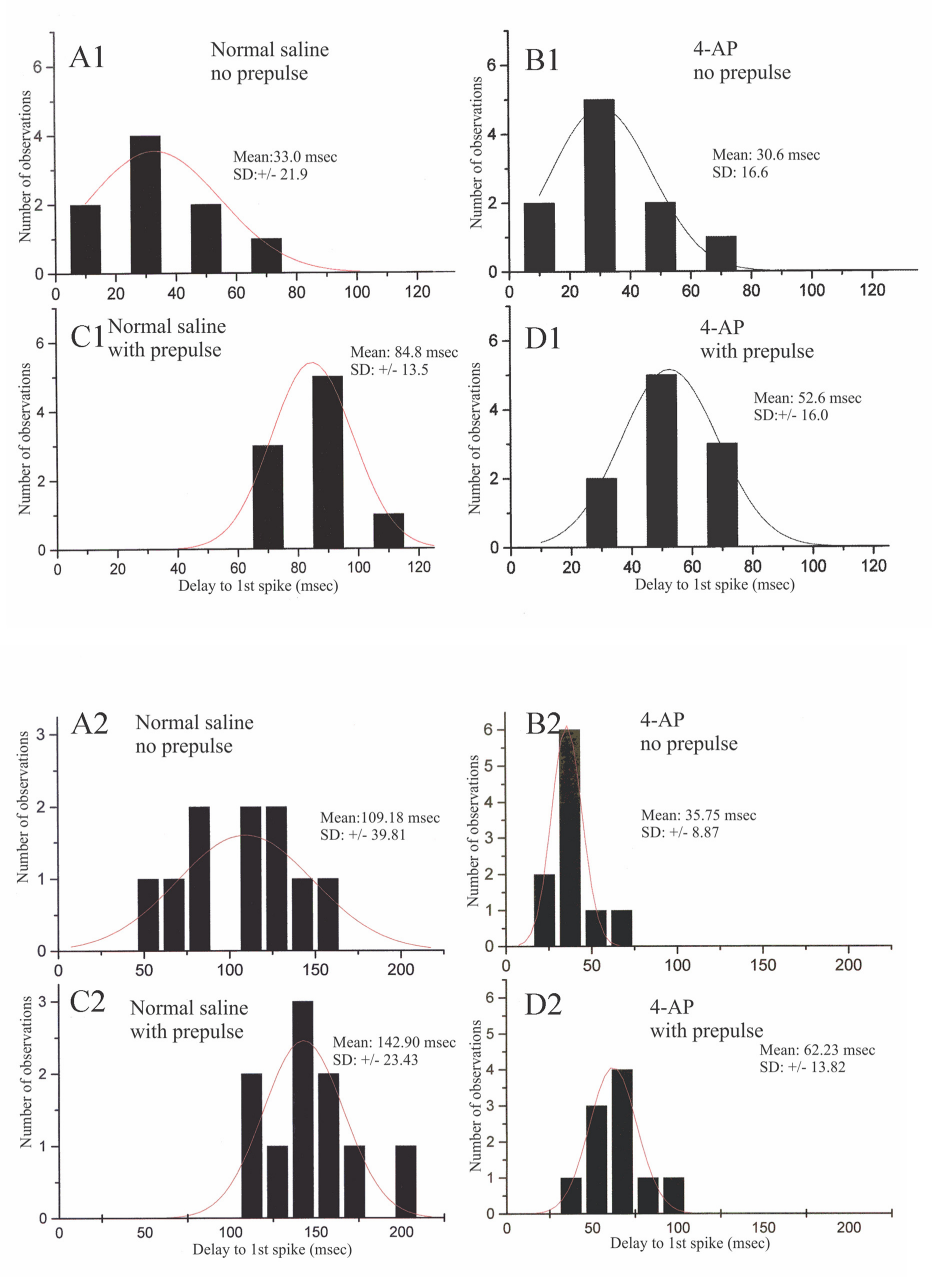
Frequency histograms of data from two cells documenting prepulse latencies with and without 4-AP. Frequency histograms of delays to first spike in two parasol cells in response to depolarizing current steps without (A1, A2) and with (C1, C2) a preceding hyperpolarizing pulse. Data in A1, A2, C1 and C2 were recorded in normal saline; identical tests were run in the two cells (B1, B2 and D1, D2) while they were in saline + 1mM 4-AP, which significantly reduced the post-hyperpolarization delay to first spike.

**Fig 9:**
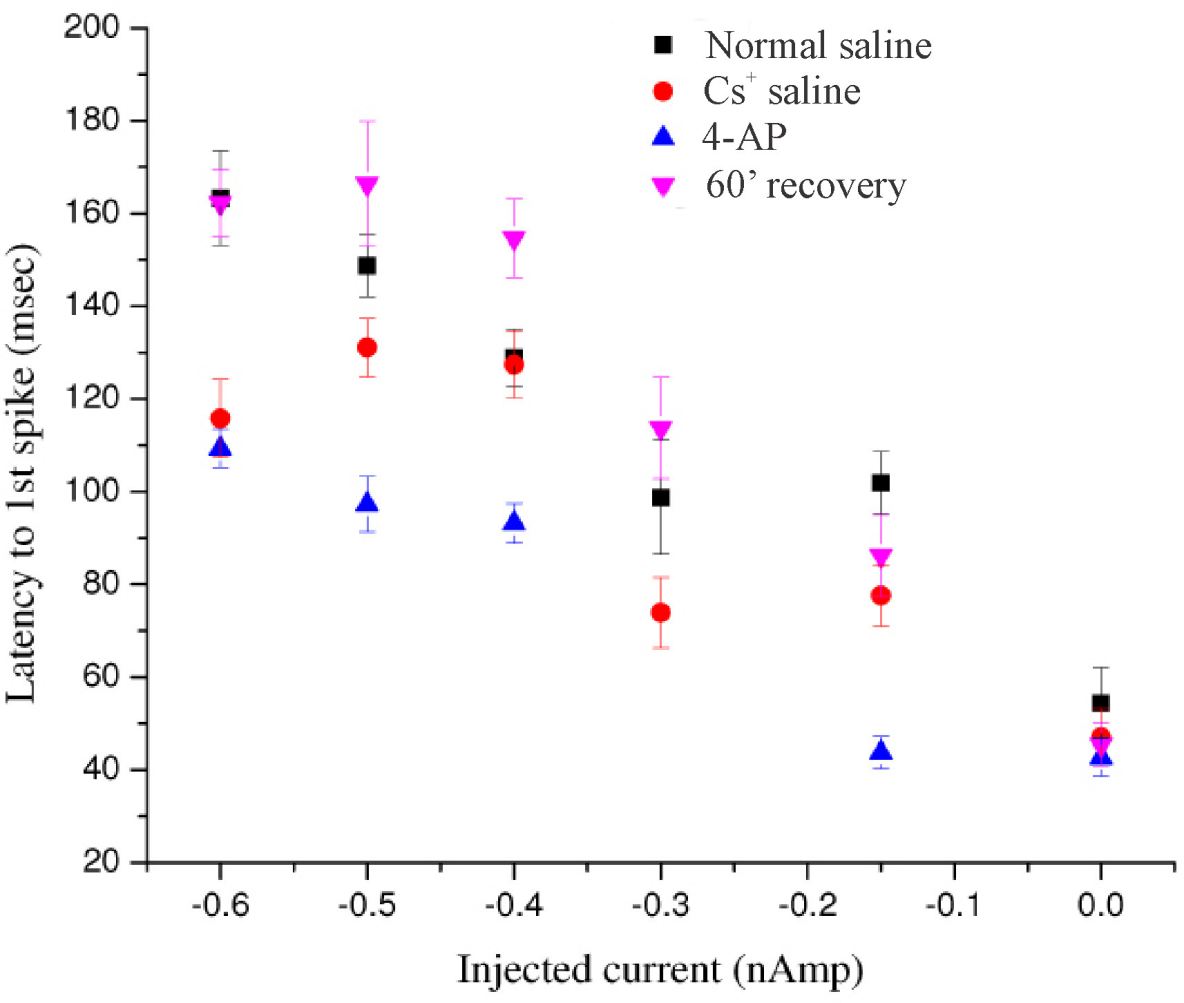
Latency to first spike data from a parasol cell under various conditions and in response to different levels of injected current. Delays to initial spike, under various experimental conditions, in response to constant depolarizing current steps immediately following 2-sec hyperpolarizing current pulses of varying amplitudes. Each data point is the mean of 10 repetitions at that current intensity, +/- one standard error. Black squares, response profile in normal saline; red circles, saline containing 5 mM CsCl; blue triangles, responses in saline with 5 mM CsCl and 1mM 4-AP; magenta triangles, responses after 90 min recovery in normal saline.

In some stomatogastric neurons of the crab (e.g., the lateral pyloric motorneuron, [32] and the lobster I_h_ is accompanied by the transient voltage-dependent potassium current I_A_. I was interested in determining whether this rapid depolarization-activated outward current is present in crayfish parasol cells that also exhibit the hyperpolarization-activated inward current. Accordingly, in seven parasol cells from different preparations, I sought evidence for I_A_ by a de-inactivation paradigm previously used in the dorsal gastric neurons of lobster STG by Tierney and Harris-Warrick [11]. Six of the seven cells tested provided evidence for the presence of I_A._ In the present paradigm, a parasol cell was hyperpolarized for 2 seconds prior to stepping the membrane potential to a constant depolarized level. The time delay to first post-hyperpolarization spike was then compared with the first-spike delay following a depolarizing step in the absence of prior hyperpolarization. Fig 6 shows specific details of the experimental protocol, including effects of the specific I_A_ blocker, 4-aminopyridine (4-AP), which promoted bursting and reduced the post-hyperpolarization delay. In Fig 7, responses following the hyperpolarization prepulse are shown in greater time detail in another preparation. In normal saline the post-hyperpolarization delay to first spike was significantly longer compared to that for the depolarization step alone; furthermore, treatment with 1 mM 4-AP reduced this delay in a reversible manner, suggesting that at least part of the post-hyperpolarization delay was due to the presence of I_A_. In parasol cells, delay measurements are made difficult due to the presence of the periodic background depolarizations which arrive at unpredictable times with respect to the end of the conditioning prepulse. The absolute level of membrane potential just prior to the depolarizing step was, therefore, not precisely controllable. Accordingly, I took the mean of the responses to ten repetitions of the several hyperpolarizing steps in each treatment regimen. The data in Fig 8, from two different cells, indicate clear differences in delay to first spike due to a depolarizing pulse alone, 33 and 109 msec, respectively as compared to the same depolarizing pulse immediately following a hyperpolarizing conditioning prepulse, 84 and 142 msec (p = 0.01). Additionally, statistically significant reductions in delay to first spike were seen in the same preparations between the 4-AP treated and untreated control trials. In four parasol cell preparations several different levels of hyperpolarization were used to record their effects on the delay to first spike at each of five levels of injected current in the different experimental treatment situations. Initial measurements were made in saline that contained 10mM CsCl to block any confounding effects of I_irk_ in response to the hyperpolarizing prepulse. The same sets of current injections were then made in saline containing both 10 mM Cs^+^ plus 1 mM 4-AP, to determine its effects on post-hyperpolarization spike delay. Fig 9 shows the results from one preparation. Current injections of – 0.15, -0.3, -0.4, -0.5, and – 0.6 nA generated different hyperpolarizing voltage steps in Cs^+^ saline, which resulted in increasing delays to the first post-hyperpolarization spike compared to the latencies with no hyperpolarizing prepulse. In saline containing both Cs^+^ and 4-AP, the parasol cell showed a reduction in the delay to the first spike following conditioning pulses generated by each level of injected current, again suggesting that I_A_ was responsible for at least part of the increased delay following the hyperpolarizing prepulse.

**Fig 10:**
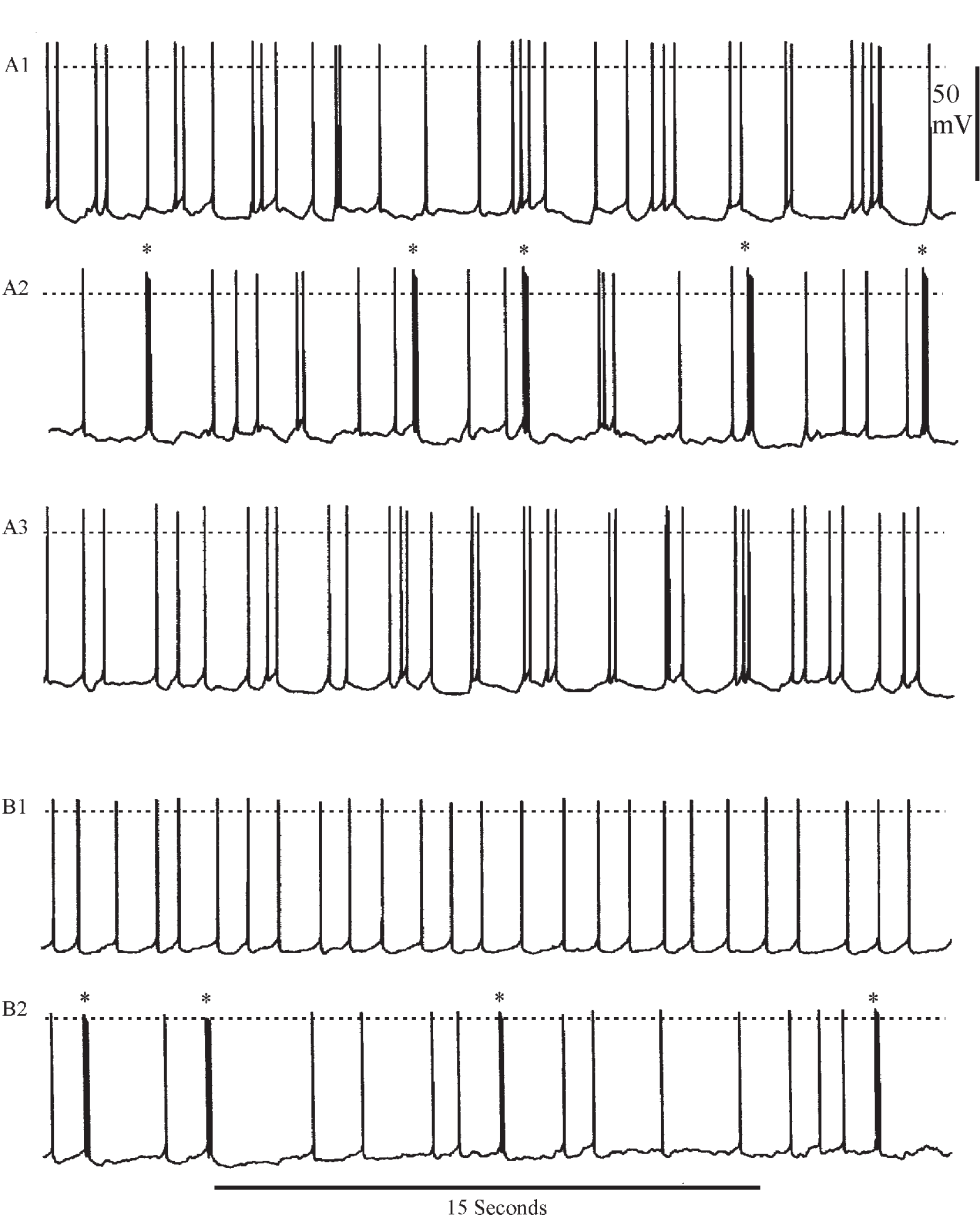
Increased spontaneous bursting in saline with 4-AP. Effects of 4-AP upon background activity in two parasol cells. A1, background activity in normal saline. A2, spontaneous bursting (*) in the presence of 1 mM 4-AP. A3, recovery in normal saline. B1, different parasol cell in normal saline. B2, following exposure to saline plus 1mM 4-AP. Bursts indicated by (*). Recording situation was lost immediately following return to normal saline. Dashed lines indicate zero membrane potential.

The experiments with 4-AP suggested that blocking I_A_ enhanced the tendency for parasol cells to generate spike bursts. Accordingly I tested 4-AP on two additional preparations to observe its effects upon background activity. Fig 10 shows the results of these experiments. Within five minutes of application, 1 mM 4-AP caused cells that previously had a low rate (< 1 burst/minute) of spontaneous bursting to generate bursts approximately every five seconds. The increased burst activity, which reversed completely in normal saline, occurred in the absence of any apparent change in the mean level of membrane potential at the recording site in the basal branches.

## Discussion

Parasol cells are conditional bursters and they generate rhythmic trains of spike bursts when driven by suitably strong sensory inputs. The generation of such burst trains and their inter-burst frequency may be partially regulated by pacemaker currents. I undertook the present study to determine the possible presence of the pacemaker currents I_h_ and I_A_ within the parasol cell population. Data presented in this paper suggest that about half of parasol cells in neuropil II of the crayfish hemiellipsoid body exhibit a hyperpolarization-activated current. The primary evidence for this current is the prominent and persistent voltage sag that develops several hundred milliseconds following the onset of a suprathreshold hyperpolarizing current injection. The sag is voltage-dependent and achieves a steady plateau, presumably reflecting the non-inactivation of the underlying current. In crustacean and other preparations [26] the hyperpolarization-activated current I_h_ is blocked by extracellularly-applied Cs^+^ ions. Cs^+^ ions were very effective in blocking the voltage sag to hyperpolarizing currents in crayfish parasol cells; however, ZD7288, a compound that is a more selective blocker of I_h_ in other crustacean neurons [27, 33] was not effective in blocking the voltage sag in parasol cells, although Cs^+^ ions tested on the same cells did so. I therefore tested the susceptibility of the hyperpolarization-induced voltage sag observed in parasol cells to saline containing either 10 mM CoCl_2_ or 10 mM TEA-Cl. Divalent cations and TEA are effective blockers of a hyperpolarization-activated inward rectifier potassium current, I_irk,_ in other invertebrate [29] and vertebrate [28] preparations; the presence of either CoCl_2_ or TEA in the saline quickly and reversibly blocked the hyperpolarization-activated voltage sag in crayfish parasol cells. The preponderance of these data, by comparisons with other invertebrate and vertebrate preparations, therefore, strongly suggest that the hyperpolarization-induced voltage sag observed in crayfish parasol cells is generated by an inward rectifier potassium current, I_irk_, and is not I_h_.

Parasol cells exhibit a prominent post-burst hyperpolarization that could be wholly or partially generated by the transient depolarization-dependent potassium current, I_A_ [7]. In the absence of voltage clamp analysis on isolated membrane currents, I inferred the presence of I_A_ in parasol cells by using a de-inactivation paradigm that has previously been employed in other crustacean [33, 11] and molluscan [34] central neurons. Using this technique and corroborative pharmacological experiments, data gathered from several (n = 7) parasol cells suggest that I_A_ is indeed present in these neurons. Within the range of hyperpolarizing pre-pulses tested, increased hyperpolarizing levels caused increasing delays to the first spike generated by a subsequent standard depolarizing current steps. Furthermore, there were statistically significant reductions in the delay to first spike using comparable hyperpolarizing pre-pulses in preparations treated with 4-AP, a specific blocker of I_A_.

### Role of intrinsic pacemaker currents in other preparations

Voltage-dependent intrinsic pacemaker currents, I_A_ and I_h_, are both present in many of the motor neurons of the crustacean STG, but are expressed to varying extents [11, 35, 14]. In the pyloric dilator neurons, not only are I_A_ and I_h_ co-expressed, but the expression appears to be co-regulated, since over-expression of the *shal* gene (which encodes I_A_) in these neurons leads to an apparent, compensatory increase in the expression of I_h_ [36, 37]. I_h_ and I_A_ both appear to play a modulatory role in generation of the pyloric motor pattern. In the dorsal gastric motor neuron of the STG, where it is co-expressed and co-regulated with the transient voltage-dependent potassium current I_A_, I_h_ contributes to the resting membrane potential, off-setting a calcium-dependent current in the opposite direction; blockade of I_h_ in these cells leads to a small (2–4 mV) increase in resting potential in TTX-poisoned preparations [38, 39]. In the lateral pyloric motor neuron of the crustacean STG, I_h_ is among the subthreshold ionic currents regulating burst generation, participating in the postinhibitory rebound that leads up to the next burst [14, 17].

In the medicinal leech, where the heart rate cycle period is controlled by a 8 neurons in ganglia 1–4 (the heartbeat oscillator), I_h_ comprises a critical inherent neuronal current that allows paired oscillator neurons to recover from mutual inhibitory interactions during each cycle [4]. Blocking I_h_ with CsCl in single isolated leech ganglia leads to continuous firing of the pacemaker neurons and, thus, complete disruption of the rhythm. Furthermore, the sensitivity of the leech heart rate to temperature changes can be accounted for by changes with temperature in both the activation kinetics and amplitude of I_h_ in the heartbeat oscillator neurons [4].

In the mammalian respiratory network, 86% of type 2 pacemaker neurons examined showed evidence for I_h_, implicating a role for this current in the generation of the respiratory pacemaker [8].

Only little more than half of the crayfish parasol cells I tested exhibited a hyperpolarization-activated voltage sag, even when tested up to 20 minutes after initial penetration by the recording electrode and the resting potential level had stabilized. Furthermore, the extent of the voltage sag produced by different levels of hyper-polarization itself varied in different cells. These observations may indicate that the expression of channels mediating hyperpolarization-activated current in any parasol cell is variable across the population, as has been found for the case for I_h_ in other crustacean preparations [40]. Differences in the expression pattern of ion channels among functionally homogeneous neurons is difficult to explain; nonetheless, the variability in expression from cell to cell may be individually compensated by modifications in the expression of other ion channels, thereby avoiding major compromises in neuronal function [41, 36]. When it was present, I found that I_irk_ contributes only modestly, if at all, to the normal physiological activity of parasol cells. For example, there was no unequivocally consistent effect of Cs^+^ ions on the mean resting potential level in crayfish parasol cells. In cells bathed in otherwise normal saline, the addition of 10 mM CsCl nominally raised the mean resting potential in only two of the eight cells examined. In the two preparations which were treated with both TTX and 10 mM CsCl, the resting potential was only slightly increased, by an average value of only 1.44 mV. Such small overall changes would be hard to measure accurately in cells bathed in saline without TTX, because variations in membrane potential due to the background synaptic activity tend to obscure such small-amplitude modulations. However, blockade of the background activity using low-Ca^2+^, high-Mg^2+^ saline, resulted in membrane depolarization and continuous spiking in parasol cells [22]; Mellon, unpublished observations). These observations raise the possibility, therefore, that a calcium-dependent outward potassium current, I_K(Ca)_, may make nominal contributions to the parasol cell resting membrane potential, and, just as in the dorsal gastric motor neurons of the STG, this could be offset by I_irk_.

The functional significance of hyperpolarization-activated inward currents can be difficult to determine in other preparations as well, where their presence is indicated by hyperpolarizing current injection. In type 2 inspiratory neurons of the mammalian respiratory network, blockade of I_h_ with either CsCl or ZD 7288 caused little change in membrane potential; nonetheless, the respiratory frequency is increased in the presence of these blockers, possibly due to inactivation of other pacemaker currents as a direct result of the depolarization induced by I_h_ [42]. This explanation is considerably weakened, however, because in high concentrations of extracellular potassium ions—a treatment which should have depolarized the relevant network components—neurons still responded to blockers of I_h_ by an increased rhythm frequency. A more plausible explanation for the action of cesium and ZD 7288 is that removal of the conductance responsible for I_h_ reduces the electrical leakiness of the respiratory pacemaker neurons, making them more responsive to excitatory currents.

### Role of I_A_ in crustacean preparations

In other rhythmic crustacean neurons I_A_ contributes to the regulation of the membrane potential trajectory between bursts; post-spike and post-burst hyperpolarizations reset the sensitivity of I_A_ to subsequent depolarizations, slowing the rise to threshold for the next burst [11,14]. Furthermore, in a number of such cells, I_A_ is subject to modulatory influences of biogenic amines. For example, in the pyloric network of the crustacean STG, the neuromodulator dopamine can phase-advance burst generation and enhance intraburst spike frequency, respectively, in the anterior burster neuron by shifting the I_h_ activation curve in a depolarizing direction [17], and by reducing both transient (presumably I_A_) and prolonged (I_K_^+^_(v)_) depolarization-activated outward currents [15]. Serotonin similarly reduces I_A_ in the anterior burster and inferior cardiac neurons of the STG, enhancing their firing [43]. In crayfish parasol cells, blocking I_A_ with 4-AP markedly increases the tendency for these neurons to generate spontaneous bursts, and sensory stimulation frequently generated burst trains, suggesting that, as in other rhythmically active cells, I_A_ plays a regulatory role in burst generation. Although serotonin also promotes spontaneous bursting in parasol cells (De F. Mellon, unpublished observations), the effects of this and other biogenic amines on intrinsic pacemaker currents in parasol cells has yet to be established.

While data presented in this paper suggest that many, perhaps a majority, of parasol cells in the crayfish lateral forebrain possess the pacemaker currents I_irk_ and I_A_, the manner in which they are integrated with the periodic synaptic background activity has not been determined. As with cells of the STG, the mechanisms by which these currents contribute to the activity of parasol cells under normal conditions are subtle; I_irk_ may modify the excitability of these neurons by maintaining the membrane potential at a level slightly more depolarized than it would be otherwise, while I_A_ may regulate the initiation of bursts and burst trains by reducing the rate of rise of the depolarizing events that would otherwise trigger them, possibly through the prominent post-burst hyperpolarization that is exhibited in all parasol cells. Until more is understood about the ionic mechanisms and channel kinetics that generate spike bursts in these crayfish brain neurons, however, our understanding of the role played by these two intrinsic pacemaker currents will remain clouded.

## Supporting Information

S1 Fig Experimental log entry 5-5-04 documenting reduction of voltage sag by 10 mM tetraethyl ammonium Chloride in two parasol cells. (TIF)

S2 Fig Logbook entry 5-6-04 documenting reduction of voltage sag in 10 mM TEA chloride and 10 mM cobalt chloride. (TIF)
