# Supplementary figures and images for: Electrophysiological Evidence for Intrinsic Pacemaker Currents in Crayfish Parasol Cells
Source: PLoS One. 2016 Jan 14;11(1):e0146091. doi: 10.1371/journal.pone.0146091 (PMC4713199; doi:10.1371/journal.pone.0146091)

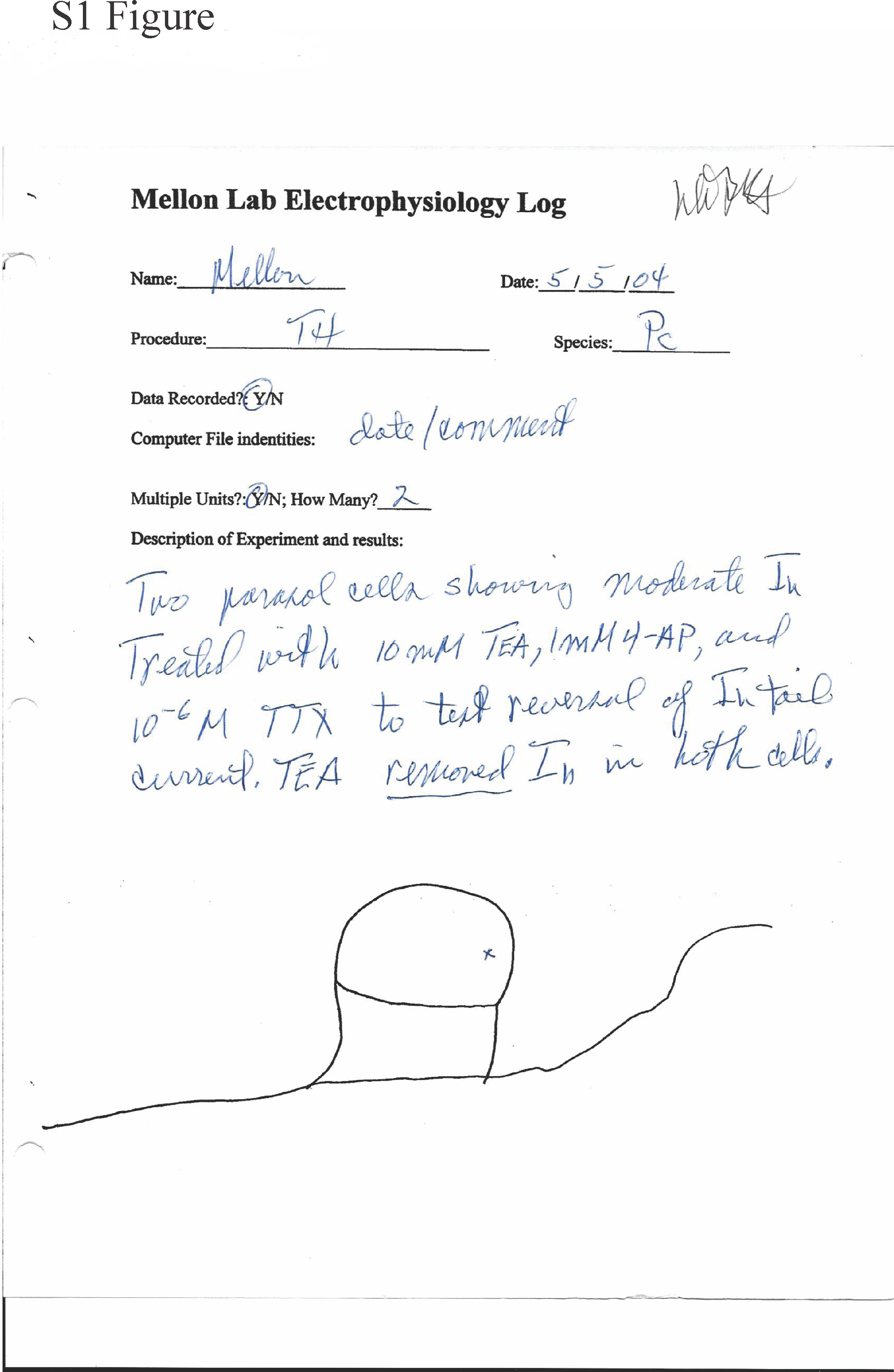

Supplement: S1 Fig — (TIF) [file pone.0146091.s001.tif]

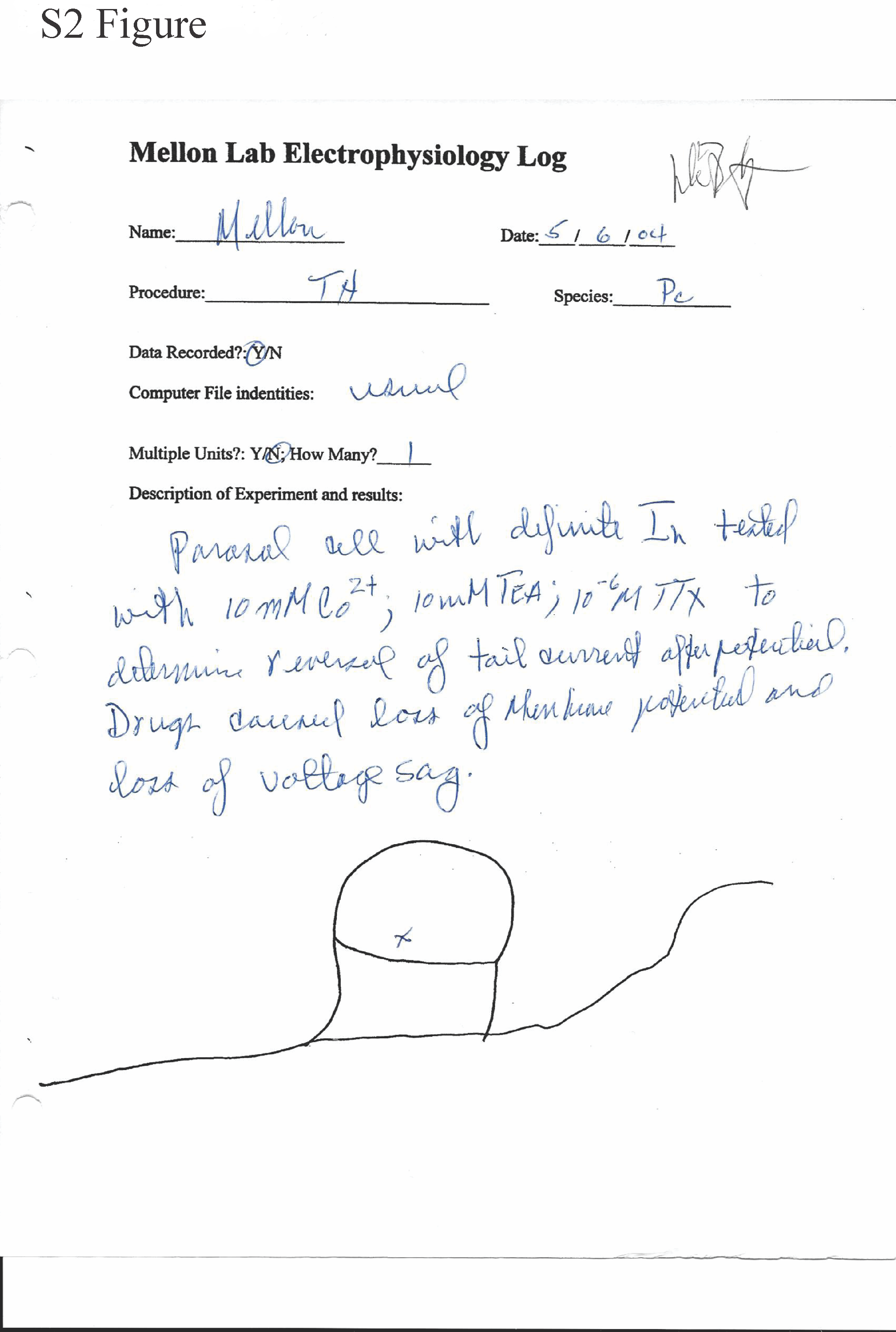

Supplement: S2 Fig — (TIF) [file pone.0146091.s002.tif]
